# The control of alternative splicing by SRSF1 in myelinated afferents contributes to the development of neuropathic pain

**DOI:** 10.1016/j.nbd.2016.09.009

**Published:** 2016-12

**Authors:** Richard P. Hulse, Robert A.R. Drake, David O. Bates, Lucy F. Donaldson

**Affiliations:** a Cancer Biology, School of Medicine, University of Nottingham, Nottingham, NG7 7UH, United Kingdom; b School of Physiology and Pharmacology, University of Bristol, University Walk, Bristol BS8 1TD, United Kingdom; c School of Life Sciences and Arthritis Research UK Pain Centre, University of Nottingham, Nottingham NG7 7UH, United Kingdom

**Keywords:** VEGF-A, vascular endothelial growth factor – A, SRSF1, Serine Arginine-rich Splicing Factor 1, SRPK1, Serine Arginine-rich Protein Kinase 1, VEGFR2, vascular endothelial growth factor receptor 2, PSNI, partial saphenous nerve ligation injury, VEGF-A, SRPK1, SRSF1, Myelinated, Spinal cord, Neuropathic pain

## Abstract

Neuropathic pain results from neuroplasticity in nociceptive neuronal networks. Here we demonstrate that control of alternative pre-mRNA splicing, through the splice factor serine-arginine splice factor 1 (SRSF1), is integral to the processing of nociceptive information in the spinal cord.

Neuropathic pain develops following a partial saphenous nerve ligation injury, at which time SRSF1 is activated in damaged myelinated primary afferent neurons, with minimal found in small diameter (IB_4_ positive) dorsal root ganglia neurons. Serine arginine protein kinase 1 (SRPK1) is the principal route of SRSF1 activation. Spinal SRPK1 inhibition attenuated SRSF1 activity, abolished neuropathic pain behaviors and suppressed central sensitization. SRSF1 was principally expressed in large diameter myelinated (NF200-rich) dorsal root ganglia sensory neurons and their excitatory central terminals (vGLUT1 + ve) within the dorsal horn of the lumbar spinal cord.

Expression of pro-nociceptive VEGF-A_xxx_a within the spinal cord was increased after nerve injury, and this was prevented by SRPK1 inhibition. Additionally, expression of anti-nociceptive VEGF-A_xxx_b isoforms was elevated, and this was associated with reduced neuropathic pain behaviors. Inhibition of VEGF receptor-2 signaling in the spinal cord attenuated behavioral nociceptive responses to mechanical, heat and formalin stimuli, indicating that spinal VEGF receptor-2 activation has potent pro-nociceptive actions. Furthermore, intrathecal VEGF-A_165_a resulted in mechanical and heat hyperalgesia, whereas the sister inhibitory isoform VEGF-A_165_b resulted in anti-nociception. These results support a role for myelinated fiber pathways, and alternative pre-mRNA splicing of factors such as VEGF-A in the spinal processing of neuropathic pain. They also indicate that targeting pre-mRNA splicing at the spinal level could lead to a novel target for analgesic development.

## Highlights

• SRSF1 (Serine Arginine-rich Splicing Factor 1) is located in myelinated sensory afferent terminals.
• Vascular Endothelial Growth Factor-A (VEGF-A) expression regulates chronic pain at the level of the spinal cord.
• SRPK1 (Serine Arginine-rich Protein Kinase 1)-SRSF1 mediated alternative RNA splicing in the spinal cord modulates pain.

## 1 Introduction

Insults to the peripheral nervous system usually result in pain and hypersensitivity to noxious (hyperalgesia) and innocuous (allodynia) stimuli. These abnormal sensations arise due to neuronal plasticity leading to alterations in sensory neuronal excitability. These alterations include peripheral sensitization (Djouhri et al., 2006), with enhanced evoked and on-going activity in primary afferents, and central sensitization, responsible for the generation and maintenance of chronic pain. The most widely accepted model for establishment of central sensitization is that ectopic firing/increased activity in C-nociceptive afferents drives altered spinal sensory processing, particularly the processing of A-fiber inputs, resulting in secondary hyperalgesia and allodynia (pain remote from an area of damage) (Li et al., 1999, Woolf and King, 1990, Woolf, 2011) (Kramer and Doring, 2013, Torebjörk et al., 1992, Ziegler et al., 1999a). C-nociceptor changes are reported in the majority of studies of animal or human neuropathies (Ali et al., 1999, Chen and Levine, 2007, Djouhri et al., 2006, Khan et al., 2002, Kirillova et al., 2011, Serra et al., 2012, Serra et al., 2014, Shim et al., 2007, Zhu and Henry, 2012) (although not all e.g. (Chen and Levine, 2007, Khan et al., 2002)). Central sensitization can also occur through neuro-immune interactions, following injury-induced local immune cell infiltration and cytokine production/release (Uceyler and Sommer, 2008). After nerve injury there is activation of spinal glia, disruption of the blood-spinal cord barrier, and consequent infiltration of immune cells (Clark et al., 2013). These events can alter the central processing of peripheral inputs, implicated in the development of chronic pain (Geranton et al., 2009, Kim et al., 2001, Torsney, 2011b). There is, however still debate on how the processing of A or C fiber inputs is differentially regulated to form the neuronal basis of chronic pain.

During chronic pain, changes in the complement of proteins result in alterations in sensory neuron excitability, as recently demonstrated whereby expression of voltage gated potassium channels in the DRG is altered in ATF3 positive sensory neurons following nerve injury (Tsantoulas et al., 2012). Furthermore, alternative mRNA splicing allows for functionally distinct proteins to arise from a single gene. This provides a vast repertoire of actions from a limited source of transcripts, allowing for cell-specific and stimulus-induced alteration in cellular function. Targeting regulation and expression of alternative RNA transcripts, and hence proteins, has been proposed as a potential route for novel drug discovery (Tavares et al., 2015), but this has not been widely investigated with respect to nociception/analgesia.

We recently demonstrated the analgesic effect of targeting alternative mRNA splicing, by inhibition of peripheral serine-arginine rich protein kinase 1, SRPK1 (Hulse et al., 2014). SRPK1 controls phosphorylation of serine-arginine rich splice factor 1 (SRSF1), which is fundamental to the control of the vascular endothelial growth factor A (VEGF-A) family alternative splicing (Amin et al., 2011, Bates et al., 2013, Nowak et al., 2010, Nowak et al., 2008). Inactive SRSF1 is located in the cytoplasm, but when phosphorylated by SRPK1 it translocates to the nucleus. There are two VEGF-A isoform families, VEGF-A_xxx_a and VEGF-A_xxx_b (Harper and Bates, 2008) where xxx refers to the number of amino acids encoded, and a and b denote the terminal amino acid sequence. SRSF1 phosphorylation results in preferential production of the proximal splice site isoforms, VEGF-A_xxx_a (Nowak et al., 2010). Little is understood about the contribution of VEGF-A proteins to nociceptive processing. VEGF receptor-2 (VEGFR2), the principal receptor activated by both isoform families, has been implicated in nociceptive processing in animal (Grosios et al., 2004, Hulse et al., 2014, Liu et al., 2012), and clinical studies (Langenberg et al., 2011). VEGF-A isoforms and VEGFR2 are present in the spinal cord (Bates et al., 2002a), and contribute to neuroregeneration and neuroprotection (Verheyen et al., 2012).

We therefore tested the hypothesis that the SRPK1/SRSF1 system contributes to spinal nociceptive processing in rodent models of neuropathic pain, concentrating on the effects of SRPK1 inhibition, and VEGF-A_xxx_a/VEGFR2 signaling in central terminals of myelinated afferents.

## 2 Materials and methods

### 2.1 Animals

Adult male Wistar rats (total 72; 250–350 g, Harlan UK) and adult male 129Ola mice (total 20; 25–30 g inbred strain) were used. Animals were provided food and water ad libitum. All animal procedures were carried out in laboratories at the University of Bristol in accordance with the U.K. Animals (Scientific Procedures) Act 1986 plus associated U.K. Home Office guidance, EU Directive 2010/63/EU, with the approval of the University of Bristol Ethical Review Group.

### 2.2 Nociceptive behavior

Nociceptive behavioral testing was carried out as previously described (Hulse et al., 2014). All animals were habituated to both handling by the tester and the testing environment on the day prior to testing. Two days of baseline testing were carried out prior to any intervention (either drug or surgical) followed by testing post-intervention at discrete time-points as detailed in each experiment. Stimuli were applied to the partially innervated medial aspect of the plantar surface of the hind paw, an area innervated by the saphenous nerve. Mechanical withdrawal thresholds were calculated from von Frey hair force response curves. Animals were housed in Perspex holding chambers with metal mesh floors (Ugo Basile) and allowed to habituate for 10 min. A range of calibrated von Frey hairs were applied to the plantar surface of the hind paw (for a maximum of five seconds or until paw withdrawal), with a total of five applications per weighted hair. From these data, force response curves were generated and withdrawal values were calculated as the weight at which withdrawal frequency = 50%. Tactile allodynia was assessed in the metal mesh floored enclosures using a brush moved across the plantar surface of the hind paw where a withdrawal scored one, with no response zero. This was repeated a total of five times giving a maximum score of five per session. Cold allodynia: a single drop of acetone was applied to the plantar surface of the hind paw using a 1 ml syringe a maximum of five times giving a maximum score of five if the animal exhibited licking/shaking behavior in response to each application. Thermal hyperalgesia (Hargreaves test (Hargreaves et al., 1988): animals were held in Perspex enclosures with a glass floor. A radiant heat source was positioned under the hind paw, and the latency was recorded for the time taken for the animal to move the hind paw away from the stimulus. This was repeated three times and a mean value calculated for each test.

Formalin Testing: animals were habituated to glass floored testing enclosures as above. A single 50 μl injection of 5% formalin was administered to the plantar surface of the right hind paw by intradermal injection. Immediately following formalin injection, animals were placed into the testing enclosures. Time (seconds) spent exhibiting pain-like behaviors and the total number of pain-like behaviors was recorded in five minute bins for sixty minutes. Data are shown as the classical biphasic response with behavioral responses pooled for the first phase 0–15 min and second phase 20–60 min. Blinding of nociceptive behavioral studies are routine in the laboratory however where animal welfare/experimental design prohibits this, it cannot be implemented. For instance, in nerve-injured animals blinding is not possible as controls are naïve. The lack of blinding may have introduced some subjective bias into these experiments, which is in part mitigated by behavioral data is supported by the inclusion of experiments in which measurements are not subjective (e.g. in vivo noxious e.m.g. recording, expression analysis, and neuronal activation using c-fos).

### 2.3 Electromyographic experiments

A well-defined method for minimally invasive preferential selection of either C- or A- fiber mediated nociceptive pathways was used (Yeomans et al., 1996, Yeomans and Proudfit, 1996). Noxious withdrawal responses to A- and C-nociceptor selective stimulation were carried out as previously described (Leith et al., 2014, Leith et al., 2007, McMullan et al., 2004), by measurement of electromyographic activity in biceps femoris. Animals were anesthetized using isoflurane induction (4% in oxygen), and the external jugular vein and trachea were cannulated to allow maintenance of airway and anesthesia. Following surgery, anesthesia was switched to alfaxalone (~ 30 mg/kg/h i.v.), and animals were maintained at a steady level of anesthesia by continuous pump perfusion via the jugular vein for the remainder of the experiment. Bipolar electrodes were made with Teflon coated stainless steel wire (Advent Research Materials, Oxford UK) implanted into the bicep femoris. EMG recordings were amplified and filtered by a combination of in-house built and Neurolog preamplifier and band pass filters (Digitimer Neurolog System). Animals were maintained at a depth of anesthesia where a weak withdrawal to noxious pinch could be elicited for the duration of the experiment. A- and C-cutaneous nociceptors were preferentially activated to elicit withdrawal reflex EMGs using a well-characterized contact heating protocol (Leith et al., 2014, Leith et al., 2007, McMullan et al., 2004). Two different rates of heating (2.5 °C/s and 7.5 °C/s) were applied to the dorsal surface of the left hind paw as these are known to preferentially activate slow/C-nociceptors (2.5 °C·s^− 1^) and fast/A nociceptors (7.5 °C·s^− 1^) respectively. Contact skin temperature at the time of onset of the EMG response was taken as the threshold. A cutoff of 58 °C for A-nociceptors, 55 °C for C-nociceptors was put in place to prevent sensitization if no response was elicited. If a withdrawal response was not elicited, threshold was taken as cut-off + 2 °C (Drake et al., 2014). Three baseline recordings were performed before i.t. drug injection with a minimum 8 min inter-stimulus interval, and alternating heating rates, to prevent sensitization or damage to the paw. Digitized data acquisition, digital to analogue conversion, and offline analyses were performed using a CED Micro1401 Mark III and Spike2 version 7 software (Cambridge Electronic Design, UK).

### 2.4 Nerve injury model

The partial saphenous nerve ligation injury (PSNI) model was used to induce mechanical and cold allodynia, as described previously (Hulse et al., 2010, Walczak et al., 2005). Under isoflurane anesthesia (3% in O_2,_), the saphenous nerve was exposed via an incision made along the inguinal fossa region of the right hind leg. Approximately 50% of the nerve was isolated and tightly ligated using 4.0 silk suture, and the incision was closed using size 4.0 sterile silk suture.

### 2.5 Drugs and drug delivery

I.t. injections were carried out under isoflurane (4% in oxygen) anesthesia, using 0.5 ml insulin syringes (29 gauge, Terumo) in rats and mice. For i.t. administration, 10 μl injections were made in the midline of the vertebral column through the intervertebral space between lumbar vertebrae five and six. The injection was deemed to be in the correct place when it evoked a tail flick response. Rats were used for i.t. anti-VEGF-A_xxx_b experiments, as the 56/1 mouse monoclonal antibody had not been validated in mice at that time. All nociceptive behavioral testing was carried out one hour after intrathecal injection as initial experiments indicated that responses to i.t. PTK peaked at 1 h, and returned to normal by 2 h after injection.

All drugs were made up as stock concentrations and then diluted to working concentration in phosphate buffered saline (PBS) as described in each experiment. Vehicle controls were used for each drug. PTK787 (LC laboratories, USA) was dissolved in polyethylene glycol (PEG) 300/PBS, with the final PEG 300 concentration at 0.002%. ZM323881 (Tocris, UK) was made up in DMSO/PBS and given intrathecally at a final concentration of 100 nM ZM323881/0.001% DMSO. Mouse monoclonal VEGF-A_165_b antibody 56/1 (AbCam ab14994; MRVL56/1), recombinant human (rh)VEGF-A_165_A (R&D systems, UK) and rhVEGF-A_165_b (R&D Systems UK) were all dissolved in PBS. SRPIN340 (N-[2-(1-piperidinyl)-5-(trifluoromethyl)phenyl]isonicotinamide; SRPK inhibitor (Gammons et al., 2013) purchased from Ascent Scientific, Bristol, UK) was dissolved in DMSO and diluted to final concentrations in PBS (to a final DMSO concentration of 0.03%). All peptides and concentrations used have been previously shown to exert functional effects in neurons and/or other biological systems (Beazley-Long et al., 2013, Hulse et al., 2014, Oltean et al., 2014). SRPIN340 has been used in several other studies, different pathological states, and was used at a known functional concentration (10 μM), as previously described (Amin et al., 2011, Hulse et al., 2014, Mavrou et al., 2015).

### 2.6 Immunohistochemistry

Rats were terminally anesthetized with sodium pentobarbital overdose (i.p. 60 mg/kg) and were perfused transcardially with saline followed by 4% paraformaldehyde. The L3-4 segments of the lumbar enlargement, containing the central terminals of saphenous nerve neurons (Phillips and Park, 1993), and L3-L4 dorsal root ganglia were removed, post fixed in 4% paraformaldehyde for 2 h and cryoprotected in 30% sucrose for 12 h. Tissue was stored in OCT embedding medium at − 80 °C until processing. A cryostat was used to cut spinal cord (20 μm) and dorsal root ganglia (8 μm) sections that were thaw mounted onto electrostatic glass slides. Slides were washed in phosphate buffered saline (PBS) solution 3 times for 5 min per incubation, and incubated in PBS 0.2% Triton X-100 for 5 min. Sections were blocked (5% bovine serum albumin, 10% fetal bovine serum, 0.2% Triton X-100 in PBS) for 2 h at room temperature, and then incubated in primary antibodies diluted in blocking solution overnight at 4 °C. Sections were washed three times in PBS washes and incubated for 2 h in secondary antibody (e.g. biotinylated or alexafluor-conjugated; 0.2% Triton X-100 in PBS). For the third stage (i.e. streptavidin-alexfluor conjugate), incubations and washes were as described for the secondary antibody. Slides were washed in PBS 3 times prior to coverslipping in Vectorshield (H1000 or H1200 containing DAPI for nuclear staining, Vector Laboratories). Images were acquired on either Nikon Eclipse E400 and a DN100 camera or Leica TCS SPE confocal microscope using Leica application suite (Tumor and Vascular Biology Laboratories' imaging suite UoN).

Primary antibodies used were as previously reported (Amin et al., 2011, Nowak et al., 2010): anti-ATF3 (rabbit polyclonal; 2 μg/ml: Santa Cruz), anti-c-fos (rabbit polyclonal; 2 μg/ml: Santa Cruz), anti-SRSF1 (goal polyclonal; 2 μg/ml; sc-10,255 Santa Cruz), anti-vGLUT1 (rabbit polyclonal, 60 pg/ml, Synaptic Systems), anti-NF200 (mouse monoclonal; 1.4 μg/ml; N0142 Sigma-Aldrich), anti-NeuN (mouse monoclonal, 1 in 100, Millipore). Use of anti-VEGF-A and SRSF1 antibodies for both immunolocalization and immunoblotting has been previously reported (Amin et al., 2011, Bates et al., 2013). Secondary antibodies (1 in 1000 dilution and from Invitrogen unless stated): Alexafluor 488 goat anti-mouse, Alexafluor 488 chicken anti-goat, Alexafluor 555 donkey anti-goat, Alexafluor 555 donkey anti-rabbit; biotinylated anti-rabbit (Stratech Scientific), Extravidin CY3 (Sigma-Aldrich). Dorsal root ganglia neuronal cell counts were performed using ImageJ analysis to measure neuronal area (μm^2^) (Schneider et al., 2012). The saphenous nerve is approximately equally derived from lumbar DRGs 3 and 4 in rat and human (Baron et al., 1988, Phillips and Park, 1993, Zhong et al., 2003); the mean number of neurons per section was quantified from 10 non-sequential random L4 DRG sections per animal. Data are presented as the mean number of neurons per section and the experimental unit is the animal. The number of activated SRSF1-positive neurons (defined as those showing nuclear localization of SRSF1) was calculated as a percentage of total neurons as designated by size (small < 600 μm^2^, medium 600 μm^2^–120 0 μm^2^, large > 1200 μm^2^) (Tsantoulas et al., 2012). The total number of DRG neurons quantified was ~ 5000 (100 neurons per section, 10 per animal, 3 per group). Determination of SRSF1 spinal cord expression/localization was determined from 5 non-sequential random spinal cord sections per animal using Image J analysis. Images were converted to an 8-bit/grayscale image then thresholding was applied across all acquired images to determine the area of positive staining. Areas of positive staining were then quantified across all sections and groups. Colocalization was determined via coloc2 plugin in ImageJ. Controls for VEGF-A and SRSF1 immunofluorescence consisted of incubation with only secondary antibody (‘no primary’ control) or substitution of the primary antibody with a species matched IgG.

### 2.7 Western blotting

Naïve and PSNI rats (treated with i.t. vehicle or SRPIN340) were terminally anesthetized (i.p. 60 mg/kg sodium pentobarbital) and perfused with saline solution. The lumbar region of the spinal cord was extracted and frozen immediately on dry ice, then stored at − 80 °C. Protein lysates (80 μg/well) were prepared using lysis buffer (RIPA buffer, Sigma-Aldrich) with protease inhibitors (Sigma-Aldrich) and samples were homogenized. Protein extracts were stored at − 80 °C until required. Samples were run on a 4% stacking gel/12% running SDS-PAGE gel (90 V, 1 h 30 min) and transferred (wet transfer) to nitrocellulose membrane for 1 h @ 100 V. Membranes were then incubated with either α-SRPK1 (mouse; 1 μg/ml; Sigma-Aldrich), α-SRSF1 (ASF/SF2; rabbit; 0.5 μg/ml; Abcam), α-SRSF1 (ASF/SF2; mouse; 0.5 μg/ml; SantaCruz), α-Actin (SantaCruz; 2 μg/ml) α-VEGF-A_165_b (mouse; 4 μg/ml; Abcam), α-pan-VEGF-A (rabbit; Santa Cruz A20; 2 μg/ml) or α-tubulin (mouse; 1 in 4000; Sigma-Aldrich) antibodies and visualized with Femto chemoilluminescence kit (exposure between 1 s and 1 min, Thermo Scientific) or Licor IRdye secondary antibodies (as previously reported (Amin et al., 2011, Gammons et al., 2013, Nowak et al., 2010)).

### 2.8 Statistical analysis

All data are represented as means ± SEM. Data were extracted and analyzed using Microsoft Excel 2010, Graphpad Prism v6 and ImageJ (Schneider et al., 2012). Nociceptive behavioral analyses were between-subjects designs comparing effects of drugs by two way ANOVA with post-hoc Bonferroni tests. In those experiments involving intrathecal and intraperitoneal administration of drugs in naïve animals, both hind paws were included in the analysis as replicates. EMG experiments used a within-subjects design and immunofluorescence experiments a between-subjects design with the effects of drug treatment compared to baseline values using one-way ANOVA with post-hoc Bonferroni tests. Immunofluorescence analysis of spinal cord (c-fos quantification) was taken from entirety of dorsal horn. DRG (SRSF1 + ve) and spinal cord (c-fos) neuron counts were ascertained from multiple representative images, at least 10 per animal and the mean value of those 10 calculated. Coloc2 analysis (Image J plugin) was used to ascertain the pixel intensity spatial correlation (co-localization) of SRSF1 and vGLUT1 staining in the spinal cord. This provides an automated measure of the correlation of pixel intensity for the two independent immunofluorescence channels for each sample, given as the Pearson's correlation co-efficient (Costes et al., 2004, Li et al., 2004). Western blot analyses of SRSF1 and VEGF-A family expression were determined from ImageJ densitometry analysis (gel analysis plug in) and compared using Mann Whitney U tests. All F test statistics are described as a column factor with reference to drug/experimental grouping. NS designates not significant.

## 3 Results

### 3.1 SRSF1 is predominantly expressed in myelinated neurons in rats

**Fig. 1:**
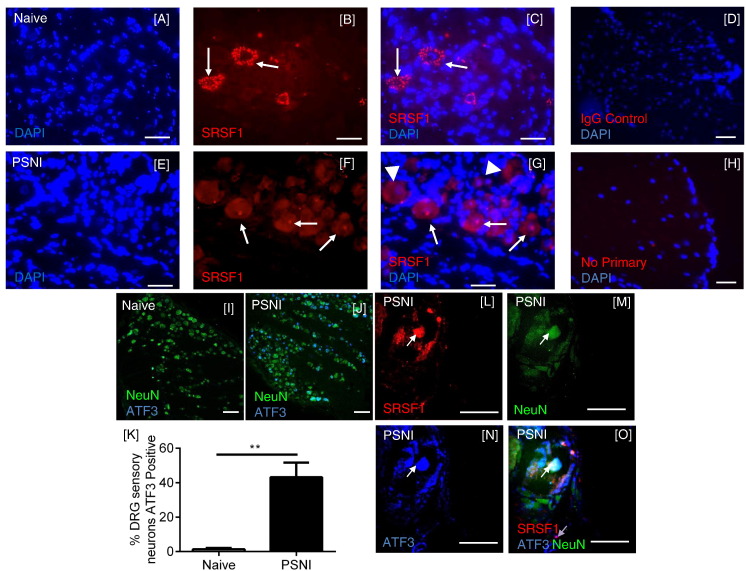
SRSF1 expression and activation in DRG sensory neurons following PSNI injury. [A–C] SRSF1 (Red) was expressed in the cytoplasm (not co-localized with DAPI) of the DRG sensory neurons in naïve animals. [D] Replacement of the primary antibody with a species matched IgG control DRG image resulted in no staining. [E–G] SRSF1 was co-localized with nuclear stain DAPI in DRG sensory neurons following PSNI injury (arrows). In some neurons cytoplasmic SRSF1 is still evident (arrowheads). [H] Omission of the primary antibody resulted in no staining. [I & J] Representative examples of ATF3 expression in NeuN-co-labeled DRG sensory neurons in [I] naïve and [J] PSNI animals. [K] The number of ATF3 positive DRG neurons was significantly increased in the L4 from PSNI animals (unpaired *t*-test, n = 5/group). [L–O] High magnification representative images of SRSF1/ATF3/NeuN co-labeled DRG neurons. (white arrows). **p < 0.001. Scale bars = 50 μm low magnification and 20 μm high magnification.

SRPK1 and SRSF1 are key factors in the control of VEGF-A_xxx_a preferential splicing particularly in disease (Amin et al., 2011, Nowak et al., 2010). SRSF1 is expressed in the cytoplasm of dorsal root ganglia (DRG) neurons in naïve animals (Hulse et al., 2014)(Fig. 1A–C). Upon activation (phosphorylation), SRSF1 is known to translocate from the cytoplasm to the nucleus (Amin et al., 2011, Nowak et al., 2010), where it is involved in pre-mRNA processing. Following PSNI, SRSF1 immunoreactivity in sensory DRG neurons was found to be nuclear (Fig. 1E-G) in some but not all neurons. Matched IgG (Fig. 1D) and omission of primary antibody (Fig.1H) controls showed no signal. PSNI injury induces activating transcription factor 3 (ATF3) expression in injured DRG sensory neurons (Beazley-Long et al., 2013). There was an increase in ATF3-positive DRG neurons after PSNI (Fig. 1I–K), with 43% of DRG neurons expressing ATF3 post-PSNI compared to only 1% in naïve animals (Fig. 1K). After PSNI, all nuclear localized SRSF1-positive (Fig. 1L) DRG neurons (Fig. 1M) were also ATF3 positive (Fig. 1N), indicating nuclear SRSF1 was exclusively found in damaged neurons (Fig. 1O). This represents that 45% of ATF3 -positive neurons were also SRSF1 positive, with the remaining 55% of ATF3 positive neurons negative for SRSF1.

**Fig. 2:**
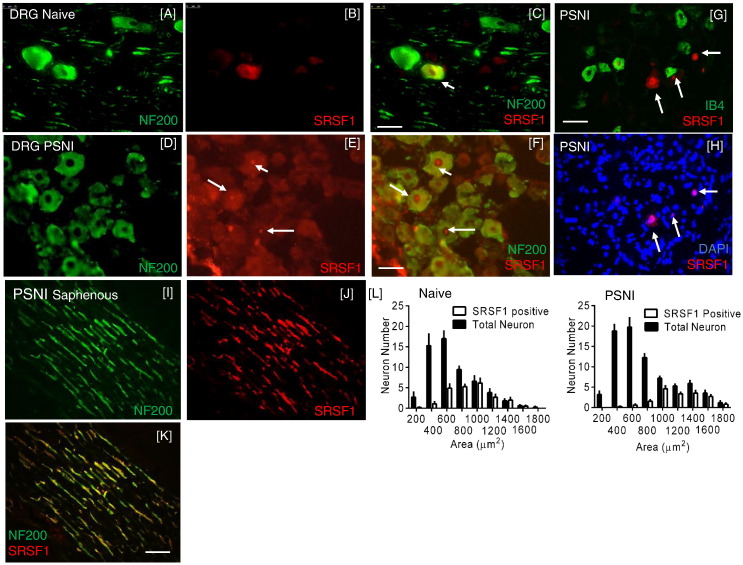
SRSF1 expression in NF200 sensory neurons. [A–C] SRSF1 expression in the cytoplasm of NF-200-positive L4 dorsal root ganglia neurons in the naïve animal. [C] Note the clear cytoplasmic localization of the SRSF1 (arrows). [D–F] Following PSNI, clear SRSF1 nuclear translocation was evident in the NF200 positive neurons (arrows in F). [G] SRSF1 was not expressed in IB4 positive dorsal root ganglia neurons, [H] though SRSF1 is co-localized with nuclear marker DAPI. [I–K] SRSF1 was also localized to NF200-rich sensory nerve fibers of the PSNI saphenous nerve. [L] Quantification of the SRSF1 positive and total number of sensory neurons in the dorsal root ganglia by cell cross-sectional area (μm^2^) in naïve and PSNI injured rats. Scale bars = 50 μm. N = 5 per group.

SRSF1 was expressed predominantly in the cytoplasm of 96% of larger (cross sectional area > 1200 μm^2^) neurofilament-200 (NF200) positive DRG neurons in naïve animals (Fig. 2A–C, L), and 71% of medium (area 601–1200 μm^2^) neurons, but was in only a small proportion (14%) of neurons of area < 600 μm^2^ (small, < 30 μm diameter). NF200 is a marker for myelinated neurons indicating that SRSF1 expression is principally found in the somata of A-fiber DRG neuronal population, but it was also found in peripheral sensory nerve fibers in PSNI animals (Fig. 2I–K). Following PSNI, activated (nuclear) SRSF1 co-localized with ATF3 and NF200 in DRG sensory neurons (Fig. 2D–F), The size distribution of activated (nuclear) SRSF1 in injured neurons was similar to that in natives, − 69% of large cells, 21.5% of medium cells but a small proportion (1.7%) of small neurons. In contrast, only a minority of the IB4-binding, largely unmyelinated DRG neurons from nerve-injured animals were positive for SRSF1 (Fig. 2G–H). The size distribution profile of DRG sensory neurons indicated that SRSF1-positive neurons are medium/large in size (Fig. 2L).

**Fig. 3:**
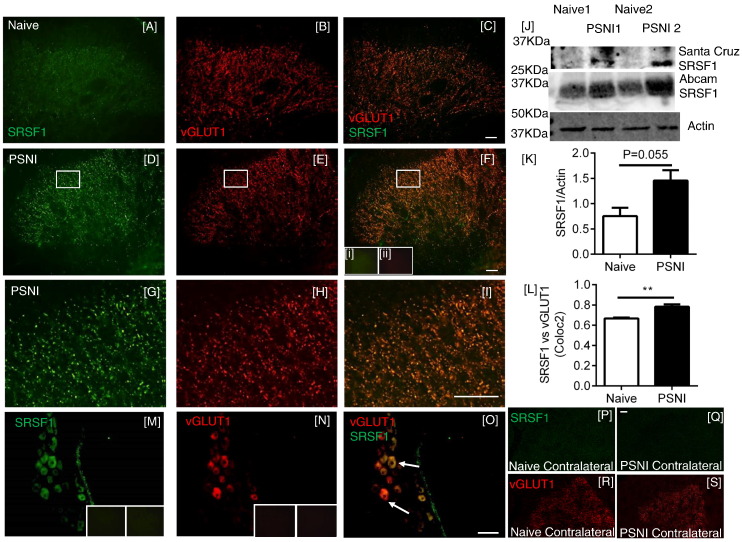
SRSF1 is expressed in myelinated central terminals in the dorsal horn of the spinal cord and increased after PSNI. [A] SRSF1 was expressed at low levels in the dorsal horn of the spinal cord in naïve animals. [B] vGLUT1 was used as a marker of myelinated sensory fiber central terminals. [C] Merged image of SRSF1 and vGLUT1. [D–F] Two days after PSNI nerve injury there was an increase of SRSF1 expression in the spinal cord, still co-localized with vGLUT1. [F] Inset images of no primary SRSF1 (i) and vGlut1 (ii) antibodies. [G–I] High power views of boxes marked in D–F. [J] Increased SRSF1 expression/localization within the lumbar spinal cord following PSNI was demonstrated by western blot with two different primary antibodies (Santa Cruz mouse monoclonal and Abcam rabbit polyclonal antibodies). [K] Quantification of increased expression post-PSNI in spinal cord vs. naïve rats (Abcam antibody, Mann Whitney *U* test, p = 0.055, n = 3) [L] using coloc2 analysis through determination of Pearson correlation coefficient, there was an increase in the degree of co-localization between vGLUT1 and SRSF1 immunoreactivity in the spinal cord following PSNI, compared to naïve (**p < 0.01 Mann Whitney test, n = 4 per group). [M] SRSF1 was expressed in DRG neurons that were [N] positive for vGLUT1, a marker of excitatory large diameter DRG neurons. [O] Overlay of vGLUT1 and SRSF1 images. [P & Q] Representative images of SRSF1 stained spinal cord sections used for analysis, showing the contralateral dorsal horn from [P] a naïve and [Q] PSNI animal. [R & S] The same images of contralateral dorsal horns showing VGLUT1 staining in [R] naïve and [S] PSNI animals (Scale bars = 50 μm).

SRSF1 immunofluorescence was also identified in the lumbar region of the spinal cord of PSNI rats, where it was co-localized with the marker of myelinated primary afferent central terminals, the vesicular glutamate transporter 1 (vGLUT1, Fig. 3A–C) (Brumovsky and Hökfelt, 2007, Neumann et al., 2008, Yasaka et al., 2014). There was an increase in SRSF1 expression in the central sensory terminals 2 days after PSNI, as assessed by immunofluorescence (Fig. 3D–I) and quantified by Western blot (Fig. 3J–K; p = 0.055). Co-localization analysis of vGLUT1 and SRSF1 staining showed a stronger colocalization in the PSNI animals (indicative of increased SRSF1 expression) in PSNI (Fig. 3L). vGLUT1 is found in large diameter myelinated neurons, and is not found in either the peptidergic or IB_4_-binding C-nociceptor populations (Brumovsky and Hökfelt, 2007, Oliveira et al., 2003). Furthermore, SRSF1 (Fig. 3M) was co-localized with vGLUT1 (Fig. 3M-O) in DRG sensory neurons. There was no SRSF1 expression in the contralateral dorsal horn of either naïve or PSNI rats, although vGLUT1 expression was evident, indicating that the increased spinal SRSF1 expression was associated with injury to peripheral neurons and not a systemic response (Fig. 3P–S).

### 3.2 Attenuation of SRSF1 mediated alternative splicing prevents A-nociceptor mediated neuropathic pain in rats

**Fig. 4:**
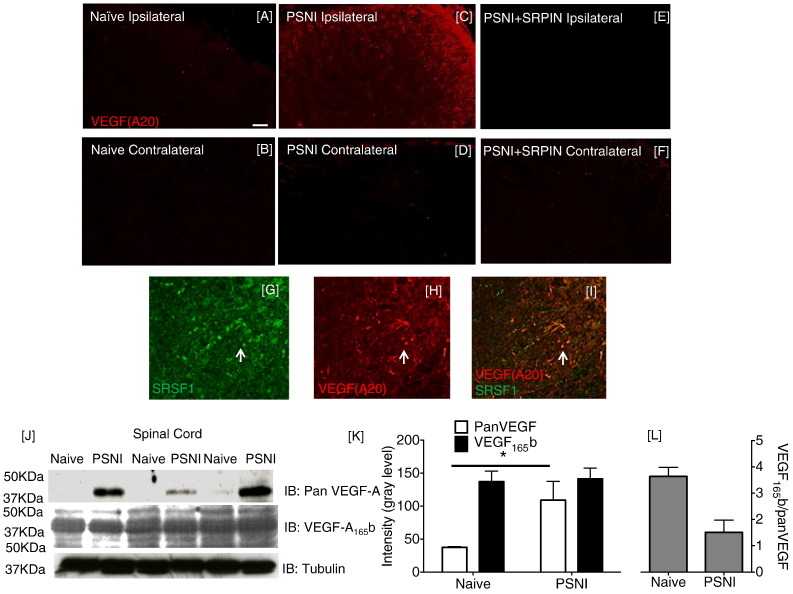
VEGF_xxx_a isoform expression increases in the spinal cord following PSNI. [A–F] Immunofluorescence of VEGF in the naïve ([A] ipsilateral [B] contralateral), PSNI ([C] ipsilateral [D] contralateral) and PSNI + SRPIN ([E] ipsilateral [F] contralateral) spinal cord (superficial dorsal horn located in top right of images) using the pan-VEGF-A antibody A20. [G–I] Co-localization of pan-VEGF-A with SRSF1 in the dorsal horn of the lumbar spinal cord (high magnification images). [J] Western blot of protein extracted from spinal cords of 6 animals, three naïve and three after PSNI. Pan-VEGF-A but not VEGF-A_165_b increased after PSNI. [K] Densitometric analysis of the Western blot showed a large increase in pan-VEGF-A expression, no increase in VEGF-A_xxx_b expression and [L] a reduction in the proportion of VEGF-A_xxx_b after PSNI versus naïve animals (one way ANOVA, Sidak post hoc test, *p < 0.05, (F(3,6) = 1.347), n = 3 per group). Scale bars = 50 μm.

The increased SRSF1 immunoreactivity in vGLUT1-positive central terminals after PSNI (Fig. 3) was accompanied by an increase in total VEGF-A expression in spinal cord (Fig. 4A–F) assessed with the pan-VEGF-A antibody A20 (Amin et al., 2011). VEGF-A was also co-localized with SRSF1 in some, but not all central terminals (Fig. 4G–I). VEGF-A_xxx_b remained unchanged in spinal cord after PSNI whereas total (pan)-VEGF-A significantly increased (Fig. 4J & K). This indicates an increase in the expression of VEGF-A_xxx_a isoforms, resulting in a decrease in VEGF-A_xxx_b as a proportion of total-VEGF-A (Fig. 4L).

**Fig. 5:**
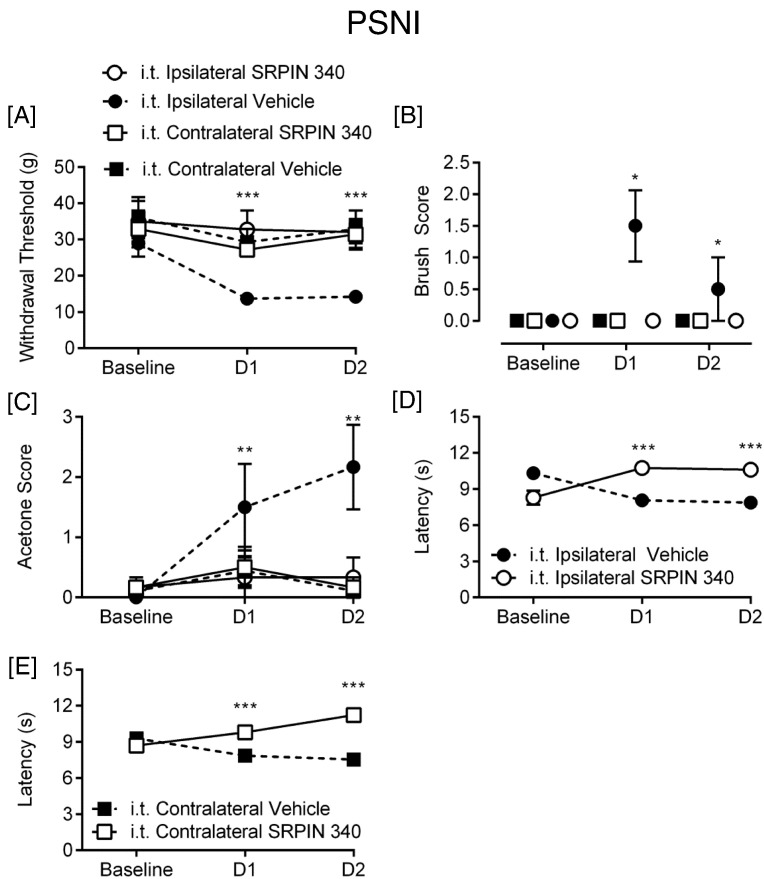
Inhibition of SRPK1 activity in the spinal cord prevents neuropathic pain. Intrathecal (i.t.) SRPIN340 treatment in rats completely prevented [A] mechanical (F test (2,20) = 3.539), [B] dynamic brush allodynia (F(2,20) = 5.526) and [C] cooling allodynia (F(2,20) = 7.8) after PSNI (n = 9, PSNI + vehicle, n = 6, PSNI + SRPIN340) in the ipsilateral hind paw. Contralateral hind-paws were not different between groups following mechanical, brush and cooling nociceptive testing. Withdrawal latencies were increased both [D] ipsilaterally (F(2,20) = 25.86) and [E] contralaterally (F(2,20) = 12.72) following i.t. SRPIN340 treatment. (*p < 0.05, **p < 0.01, ***p < 0.001 two way ANOVA with post-hoc Bonferroni tests).

**Fig. 6:**
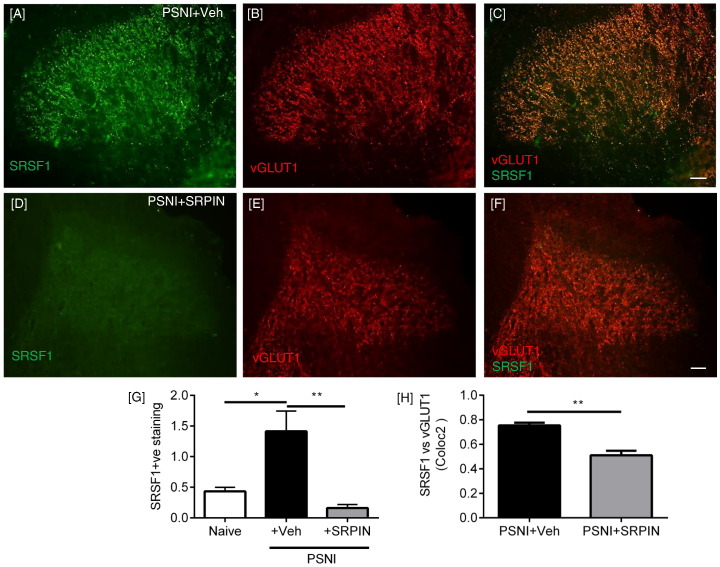
PSNI increases and intrathecal SRPIN340 reduces SRSF1 expression in the spinal dorsal horn. [A–C] SRSF1 immunoreactivity in vGLUT1-positive terminals in the spinal cord after PSNI. (C shows the co-localization of SRSF1 and vGLUT1). [D–F] Intrathecal 10 μM SRPIN340 reduced SRSF1 immunoreactivity in vGLUT1-positive terminals. [F] indicates that there is a loss of expression of SRSF1 but not vGLUT-1. [G] Quantification of SRSF1/vGLUT1 fluorescence intensity by area. PSNI increased SRSF1 staining and SRPIN340 treatment led to a reduction in SRSF1 immunostaining within the dorsal horn 2 days after PSNI (F(2,9) = 11.16, *p < 0.05, **p < 0.01 one way ANOVA with post-hoc Bonferroni test; n = 4 per group). [H] Intrathecal SRPIN 340 treatment in PSNI injured animals demonstrate a reduction in colocalization between vGLUT1 and SRSF1 compared to PSNI + vehicle group (**p < 0.01, Mann Whitney test, n = 4 per group).

**Fig. 7:**
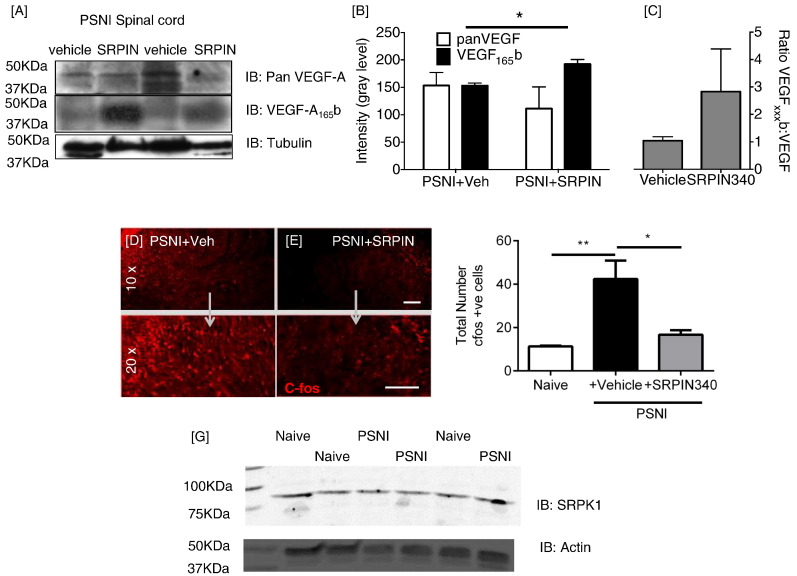
Inhibition of SRPK1 in the spinal cord following PSNI leads to reduction in VEGF-A_xxx_a expression. [A] Immunoblotting for pan-VEGF-A, VEGF-A_xxx_b and tubulin expression in spinal cord from 4 PSNI animals treated with vehicle or SRPIN340. [B] Quantification of intensity showed that the amount of VEGF-A_xxx_b increased slightly, and pan-VEGF-A reduced resulting in [C] a restoration of the VEGF-A_165_b ratio in PSNI toward that in naïve control animals (compare with Fig. 3H, one way ANOVA, *p < 0.05 Sidak test (F(3,6) = 3.529) n = 3 per group). [D–E] C-fos immunostaining in spinal cord dorsal horn in PSNI animals treated with either i.t. vehicle or SRPIN340. [F] Increased spinal neuronal activation, indicated by increased numbers of c-fos expressing dorsal horn neurons after PSNI, was blocked by PSNI + SRPIN340 treatment (one way ANOVA with post Bonferroni test, *** p < 0.001, (F(2,9) = 36.50), n = 4 per group for c-fos expression). [G] SRPK1 was expressed in the lumbar spinal cord in the naïve animal, and was unchanged post-PSNI (n = 3 per group, NS) Scale bar = 40 μm.

These results suggest that SRSF1 phosphorylation and activation at the level of the spinal cord is induced by PSNI, and is accompanied by a change of the balance of VEGF isoforms toward VEGF-A_xxx_a. As VEGF-A_165_a has been shown to be pro-nociceptive, and VEGF-A_165_b anti-nociceptive (Hulse et al., 2014), it is therefore possible that changes in SRSF1 and VEGF-A expression at the level of the spinal cord are associated with the development of neuropathic pain behaviors. SRSF1 activity is activated through phosphorylation by serine-arginine-rich protein kinase SRPK1 (Amin et al., 2011). To test the hypothesis that PSNI neuropathic pain is dependent upon SRSF1 activation, we inhibited SRPK1 in the spinal cord of rats, with intrathecal (i.t) injection of the SRPK1 antagonist, SRPIN340 (N-[2-(1-piperidinyl)-5-(trifluoromethyl)phenyl] isonicotinamide, Ascent Scientific, Bristol UK) (Fukuhara et al., 2006) (10 μM i.t. injection) at the time of nerve injury surgery (time point day 0). SRPIN340 has been used extensively to inhibit SRPK1 activity and a multitude of studies have demonstrated its involvement with controlling alternative splicing for VEGF-A isoforms (Amin et al., 2011, Mavrou et al., 2015, Nowak et al., 2010), through suppression of SR protein phosphorylation and stabilization (Fukuhara et al., 2006). SRPIN340 inhibits both SRPK1 and SRPK2 at concentrations equal or < 10 μM (Fukuhara et al., 2006), and this has been shown previously to inhibit VEGF-A_xxx_a production in vitro (Nowak et al., 2010) and in vivo (Amin et al., 2011). PSNI induced a reduction in mechanical withdrawal thresholds in the ipsilateral hind paw as expected, and this was blocked by i.t. SRPIN340 (Fig. 5A; PSNI + vehicle n = 9, PSNI + SRPIN n = 6). Tactile and cooling allodynia which also developed in the ipsilateral hind paw (Figs. 5B & C) were also inhibited by SRPIN340. Contralateral hind paws from vehicle and SRPIN340 treated groups did not differ from each other, indicating no effect of central SRPK1 inhibition on noxious processing from uninjured tissue. The PSNI model does not in itself lead to the development of heat hyperalgesia (Hulse et al., 2010), but Hargreaves latencies did increase as a result of SRPIN340 treatment compared to vehicle treated PSNI animals, both ipsilateral (Fig. 5D) and contralateral (Fig. 5E) to the nerve injury, indicating a possible contribution of SRPK1/SRSF1 in normal nociceptive processing. SRPIN340 treatment also resulted in a significant inhibition of the increase in SRSF1 immunoreactivity in the central terminals of the dorsal horn of the spinal cord induced by PSNI (Fig. 6A–H). Furthermore, the administration of SRPIN340 resulted in increased distal splice site, anti-nociceptive isoform VEGF-A_xxx_b with no overall change in total VEGF-A expression (Fig. 7A), indicating a switch from proximal to distal splice site transcripts following SRPIN treatment in peripheral nerve injury (Fig. 7B–C). Intrathecal SRPIN340 not only blocked the development of nociceptive behaviors and altered alternative splicing in the dorsal horn, it also blocked indicators of central sensitization. The number of c-fos positive neurons in the spinal cord, a marker of central sensitization (Hunt and Evan, 1987) as assessed by immunofluorescent staining (Fig. 7D), was increased after PSNI and was significantly reduced by i.t. SRPIN340 (Fig. 7E–F). SRPK1 protein expression within the spinal cord was not significantly altered following nerve injury alone (Fig. 6G).

### 3.3 VEGF-R2 activation at the level of spinal cord contributes to nociceptive processing

VEGF-A_xxx_a and VEGF-A_xxx_b differ only in their terminal 6 amino acids. The C-terminal sequence determines the efficacy of VEGFR2 signaling of the isoforms and their functional properties (Cébe Suarez et al., 2006). On binding to VEGFR2, VEGF-A_xxx_a leads to full phosphorylation and activation of VEGFR2, whereas VEGF-A_xxx_b activates only partial VEGFR2 phosphorylation, leading to receptor degradation (Ballmer-Hofer et al., 2011). VEGF-A_165_b also antagonizes VEGF-A_xxx_a binding (Woolard et al., 2004). The different C-terminal sequences also determine the anti- or pro-nociceptive effects of the VEGF-A_165_b and VEGF-A_165_a isoforms respectively (Hulse et al., 2014) but both isoforms promote neuroprotection (Beazley-Long et al., 2013, Sondell and Kanje, 1999). Our findings above show that VEGF-A alternative splicing is altered in neuropathic states (Fig. 3, Fig. 4, Fig. 5), and this is associated with pain behaviors. These results suggest that spinal cord VEGFR2 activation by different VEGF isoforms could contribute to nociceptive processing. Despite evidence from clinical studies that demonstrate an involvement of VEGF receptors in pain (Langenberg et al., 2011, McCarthy and McCrory, 2013), and experimental evidence showing that spinal VEGF levels are associated with pain (Nesic et al., 2010), there are few published findings on the effects of VEGF-A in spinal nociceptive processing. As spinal VEGF-A splicing and isoform expression, and therefore by inference VEGFR2 activation, were altered in PSNI we determined the effect of VEGFR antagonism on central nociceptive processing.

**Fig. 8:**
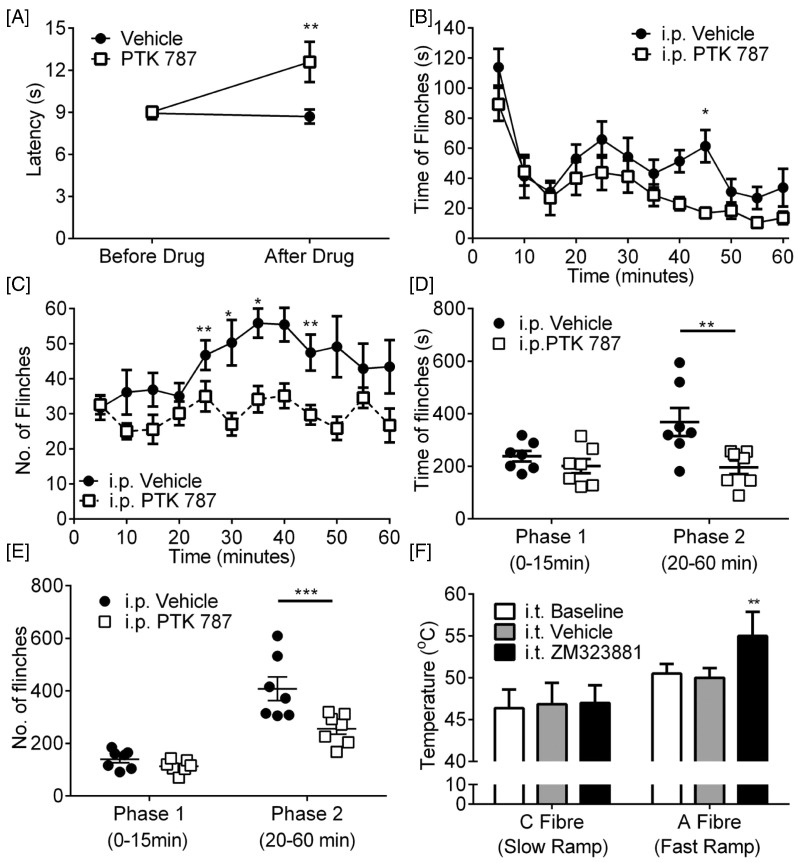
VEGF receptor 2 blockade leads to attenuation of nociceptive pain behavior in rats. [A] Intraperitoneal injection of 30 mg/kg PTK787 led to an increased withdrawal latency to heat (two way ANOVA with post-hoc Bonferroni test n = 5/group, **p < 0.01, (F(1,20) = 5.388). Intraperitoneal 30 mg/kg PTK787 attenuated both [B] time (F(11,132) = 13.39) and [C] number (F(11,132) = 4.015) of formalin-induced pain behaviors within the second phase (two way ANOVA with post-hoc Bonferroni test, *p < 0.05, **p < 0.01, n = 7/group). Area under the curve analysis of [D] duration (F(1,12) = 5.874) and [E] number (F(1,12) = 8.739) for the two phases of nociceptive behaviors shown in B & C (**p < 0.01, ***p < 0.001 two way ANOVA with post-hoc Bonferroni test). [F] Intrathecal injection of 200 nM of VEGFR2 antagonist ZM323881 led to an increase in EMG response threshold only to A-nociceptor stimulation versus baseline and vehicle groups (**p < 0.01; two way ANOVA with post Bonferroni) (n = 3–5/group).

PTK787 (or vatalanib) is a tyrosine kinase inhibitor that has non-selective inhibitory actions on VEGFR1 and 2. It is 18-fold more selective for VEGFR1 and 2 over VEGFR3, and has slight selectivity for VEGFR2 (IC_50_ < 50 nM) over VEGFR1 (IC_50_ ~ 100 nM) (Wood et al., 2000). In naïve rats, systemic VEGFR antagonism with PTK787 (30 mg/kg, i.p.) increased thermal withdrawal latencies to heat (Fig. 8A n = 5/group) indicating an analgesic effect. To determine the effect of PTK787 on one aspect of central nociceptive processing, we used the formalin test. Injection of formalin into the hind paw allows for the investigation of two distinct phases of acute nociceptive behavior. The initial phase (0–15 min) is largely mediated by peripheral nerve activation, whereas the second has both a peripheral and central component. One hour prior to formalin injection, rats were treated with either (i.p.) vehicle or PTK787. The acute phase was unaffected (0–15 min) by PTK787 treatment (Fig. 8B–E; n = 7/group). In contrast the second phase (20–60 min) was significantly reduced by systemic PTK787 treatment for both the time of flinching (Fig. 8B & D) and the number of flinches (Fig. 8C & E). These results suggest a central component of VEGFR inhibition. To determine the targets of VEGF-A/VEGFR signaling in naïve rats, given the effects of the VEGFR antagonist on the second phase of the formalin test, we recorded electromyographic nociceptive withdrawals to selective nociceptor activation. Fast heating (fast heating rates ~ 7.5 °C/s) preferentially activates myelinated A-nociceptors and slow heating activates unmyelinated C-nociceptors, both inducing a withdrawal from the stimulus. To determine VEGFR2 specific actions, ZM323881 (5-[[7-(benzyloxy) quinazolin-4-yl]amino]-4-fluoro-2-methylphenol) was used locally. ZM323881 which has sub-nanomolar potency and specificity for VEGFR2 (IC_50_ < 2 nM) (Whittles et al., 2002), with an IC_50_ > 50 μM for VEGFR1 and PDGFR (Whittles et al., 2002). I.t. ZM323881 (100 nM, specific VEGFR2 inhibitor, (Whittles et al., 2002) led to a prolonged (up to 60 min) increase in the temperature at which the rats withdrew during A-nociceptor stimulation (Fig. 8F, n = 3–5 per group). ZM323881 did not have a significant effect on C-nociceptor withdrawals (Fig. 8F). These results show that VEGFR2 signaling is mediated, at last in part, by A-nociceptor activation in the spinal cord.

**Fig. 9:**
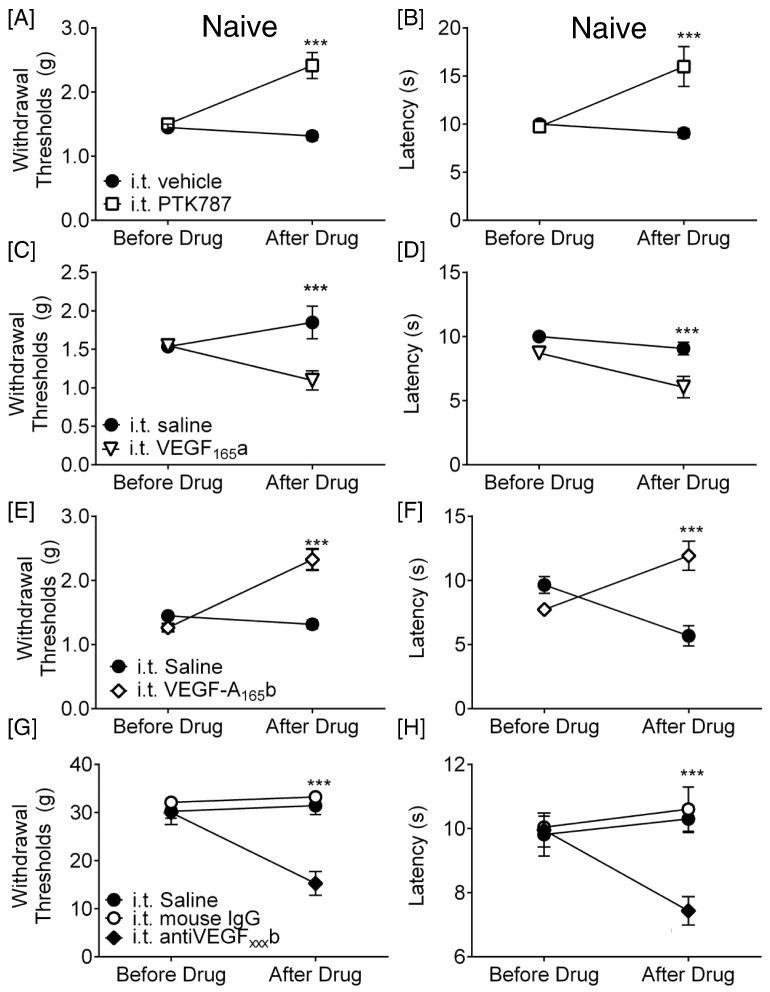
Alteration of spinal VEGFR activation attenuates nociceptive behavior in naïve mice and rats. [A] Intrathecal administration of 200 nM PTK787 increased mechanical withdrawal thresholds (F(1,10) = 12.47) and [B] increased withdrawal latency to heat in mice (F(1,12) = 8.165, n = 4/group vehicle, (8 hind paws used as replicates), n = 3/group PTK787, (6 hind paws used as replicates), **p < 0.01 two-way ANOVA with post-hoc Bonferroni test). [C] Intrathecal VEGF-A_165_a reduced mechanical thresholds (F(1,12) = 17.18) and [D] heat (F(1,12) = 18.61) withdrawal latencies in mice (n = 4/group (8 hind paws used as replicates). [E] Intrathecal VEGF-A_165_b increased mechanical thresholds (F(1,12) = 25.26) and [F] thermal (F(1,16) = 5.631) response latencies in mice (n = 4 vehicle group (8 hind paws used as replicates), n = 5 VEGF group, (10 hind paws used as replicates)). [G] Treatment of rats with a VEGF-A_165_b neutralizing antibody decreased both mechanical thresholds (F(1,15) = 18.66) and [H] thermal latencies (F(1,15) = 1.400, n = 3 group (6 hind paws used as replicates), two way ANOVA with post-hoc Bonferroni test, ***p < 0.001).

Taken together, these results are consistent with the hypothesis that the VEGF-A isoforms may have different functions in the spinal cord, as in the periphery (Hulse et al., 2014). We tested this by giving VEGF agonists and antagonists intrathecally (i.t.), and measuring pain behaviors in mice and rats. PTK787 increased both mechanical withdrawal thresholds (Fig. 9A; n = 3 mice/group, 6 hind-paws treated as replicates) and heat nociceptive withdrawal time (Fig. 9B) compared with vehicle treated mice. In contrast injection of 2.5 nM VEGF-A_165_a reduced mechanical withdrawal thresholds (Fig. 9C; n = 4 mice/group, 8 hind-paws treated as replicates) and heat withdrawal latencies (Fig. 9D), indicating a central pro-nociceptive action of VEGF-A_165_a in naïve mice. Conversely, 2.5 nM VEGF-A_165_b increased mechanical thresholds (Fig. 9E n = 4 mice group, 8 hind-paws treated as replicates) and heat withdrawal latencies (Fig. 9F) indicating a central anti-nociceptive effect. In rats, administration of a neutralizing antibody against VEGF-A_xxx_b had a similar effect to that of VEGF-A_165_a, decreasing withdrawal thresholds to mechanical stimulation (Fig. 9G; n = 3 rats group, 6 hind-paws treated as replicates) and the time taken for withdrawal from heat (Fig. 9H), indicating that loss of endogenous VEGF-A_xxx_b from the spinal cord is painful in naïve animals.

### 3.4 Attenuation of central VEGFR2 signaling leads to alleviation of neuropathic pain

**Fig. 10:**
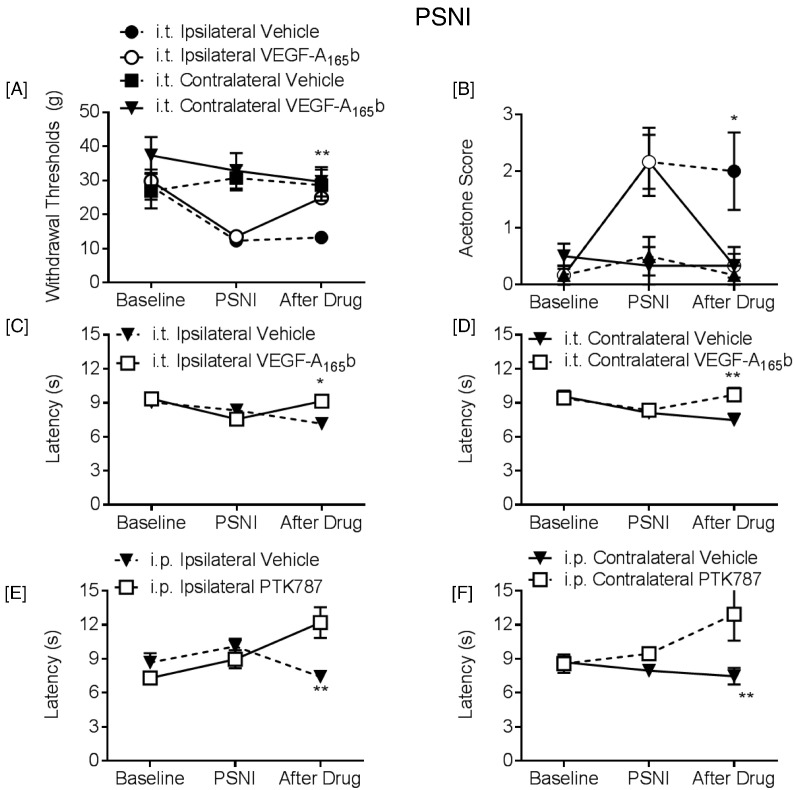
Attenuation of VEGFR2 signaling leads to alleviation of neuropathic pain in rats. Intrathecal application of VEGF-A_165_b two days after PSNI surgery abolished [A] mechanical (F(2,10) = 32.39) and [B] cooling (F(2,20) = 14.03) allodynia (n = 6 per group), and increased withdrawal latencies to heat in both [C] ipsilateral (F(2,20) = 4.201) and [D] contralateral hind paws (F(2,10) = 3.476, two way ANOVA with post-hoc Bonferroni test, *p < 0.05, **p < 0.01, ***p < 0.001, n = 6 per group). Contralateral hind-paws from both groups did not differ in nociceptive behavioral response to [A] mechanical and [B] cooling stimulation. IP 30 mg/kg PTK787 led to increased withdrawal latencies to heat in the [E] ipsilateral (F(2,12) = 2.45) and [F] contralateral limb (F(2,12) = 1.38) (two way ANOVA with post-hoc Bonferroni test, **p < 0.01, n = 4 per group).

We mimicked the effect of spinal SRPK1 inhibition by increasing the proportion of spinal VEGF-A_165_b with exogenous protein, 2 days after the onset of neuropathic pain behavior in rats. Intrathecal VEGF-A_165_b reversed both mechanical (Fig. 10A) and cold allodynia (Fig. 10B) and increased thermal withdrawal latencies both ipsilaterally (Fig. 10C) and contralaterally (Fig. 10D). IP (30 mg/kg) PTK787 led to the increase in withdrawal latencies to heat both ipsilateral (Fig. 10E) and contralateral (Fig. 10F) in PSNI injured rats.

## 4 Discussion

We show that the splicing factor kinase SRPK1 is a key regulator of spinal nociceptive processing in naïve and nerve injured animals. We present evidence for a novel mechanism in which altered SRSF1 localization/function in neuropathic pain results in sensitization of spinal cord neurons. Inhibiting the splicing factor kinase SRPK1 can control alternative splicing of VEGF-A isoforms in spinal cord, and can prevent the development of neuropathic pain.

### 4.1 Alternative splicing and pain

The development of neuropathic pain and associated neuronal excitation, results from alterations in neuromodulatory protein function, leading to sensitization of peripheral and central nociceptive systems. Both short and long term changes occur in the expression and function of ion channels, receptors, excitatory and inhibitory neurotransmitters/modulators and second/third messenger systems (Cheng and Ji, 2008, Tsantoulas and McMahon, 2014, Tsantoulas et al., 2012) leading to the regulation of neuronal excitability through modulation of excitatory and/or inhibitory networks. Many of these alterations can be attributable to altered protein expression (e.g. (Obara and Hunt, 2014, Saab, 2012). Alternative pre-mRNA splicing is a rapid, dynamic process, recognised to be important in many physiological processes, including in nociception (Kalsotra and Cooper, 2011). Such splicing of many channels and receptors particularly calcium channels, is altered in pain states (Asadi et al., 2009, Nakae et al., 2013), but prior to our studies the control of mechanisms of alternative pre-mRNA splicing had not been considered as a contributory factor in nociceptive processing (Hulse et al., 2014).

### 4.2 Inhibition of SRPK1 alleviates neuropathic pain and reduces SRSF1 activation

The splicing kinase SRPK1, a member of the serine-arginine-rich kinases, controls alternative pre-mRNA splicing of a relatively small number of identified RNAs (Hulse et al., 2014). To date, there is strong evidence for the involvement of only one of these, VEGF-A, in nociception (Hulse et al., 2014, Lin et al., 2010, Liu et al., 2012, Ropper et al., 2009, Verheyen et al., 2012). SRPK1 controls the activity of splice factor SRSF1 that is fundamental to the processing of pre-mRNA transcripts (Ghosh and Adams, 2011), their cellular localization/transport (Bjork et al., 2009), and it may also be involved in translational repression (Delestienne et al., 2010). Phosphorylation and activation of SRSF1 results in nuclear translocation in a number of cell types (Amin et al., 2011, Nowak et al., 2010). After nerve injury activated SRSF1 was only found in the nuclei of injured (ATF-3 positive) large excitatory (vGLUT1 positive) neurofilament-rich DRG neurons whereas it was found in the cytoplasm of uninjured DRG neurons. Interestingly, SRSF1 was also seen in the central terminals of myelinated neurons after injury, but was not in central terminals in naïve animals. The nuclear localization suggests that neuronal SRSF1 is activated in mRNA processing in injured myelinated neurons (Amin et al., 2011). The redistribution of cytoplasmic SRSF1 to central terminals may reflect a change in neuronal function or mRNA transport (Tripathi et al., 2012). Little is understood of this function of SRSF1 in sensory neurons, although mRNA transport is closely linked to splicing, and specific mRNA splice variants can be targeted to axons (Minis et al., 2014).

After traumatic nerve injury, injured DRG neurons (e.g. ATF3 positive) demonstrate ectopic and/or increased evoked activity. These neuronal phenomena arise due to expression changes in key mediators of sensory neuronal excitability, ultimately underlying chronic pain phenotypes (Djouhri et al., 2006, Tsantoulas et al., 2012). Local neuro-immune interactions resulting from damage to neurons alter the properties of adjacent ‘uninjured’ afferents (Djouhri et al., 2006, Tsantoulas et al., 2012), including sensitization of A-fiber afferents (Zhu and Henry, 2012), and together these drive excitability changes in the spinal cord (Costigan et al., 2009). Mechanisms such as SRPK1/SRSF1-mediated alternative pre-mRNA splicing could underpin this ‘phenotypic switch’ change in properties, for example by controlling relative expression of ion channel splice variants in damaged neurons (Asadi et al., 2009, Tsantoulas et al., 2012). Increased release of neurotransmitters and modulators from primary afferent central terminals is seen in the spinal cord following nerve injury (Gardell et al., 2003). The cellular SRSF1 redistribution also suggests that phosphorylated SRSF1 could act to transport RNAs to the central terminals in nerve injury, and hence enable translation of specific isoforms (e.g. VEGF-A_165_a) in the nerve terminals (Gardell et al., 2003). This reduction in the amount of SRSF1 present in afferent central terminals following intrathecal SRPK1 inhibition could be due to increased degradation of the SRPK1-SRSF1 complex and/or reductions in transport of mRNA to the central terminals of primary afferents.

In addition to peripheral sensitization, PSNI results in mechanical and cold hypersensitivity (Hulse et al., 2010) and central sensitization (Walczak et al., 2005). Intrathecal administration of the SRPK1 inhibitor SRPIN340 abolished pain behaviors including mechanical allodynia and hyperalgesia, and cold allodynia, and the central sensitization indicated by spinal c-fos expression. Central hyperalgesic priming of primary afferent nociceptors is dependent on local protein translation in central terminals (Ferrari et al., 2015), so we speculate that SRPK1/SRSF1 actions on RNA localization or protein translation (Bjork et al., 2009, Delestienne et al., 2010) may also contribute to this sensitization mechanism. As heat hyperalgesia was also reduced but PSNI animals did not display sensitization to radiant heat (Hulse et al., 2008, Walczak et al., 2005), this suggests that central SRPK1 inhibition not only prevents central sensitization, but also reduces activation of non-sensitized spinal nociceptive networks.

### 4.3 VEGF splicing and VEGF-dependent nociceptive processing in spinal cord

SRPK1/SRSF1 controls the splice site choice in the alternative splicing of the vascular endothelial growth factor A (VEGF-A) family, leading to increased expression of VEGF-A_xxx_a isoforms (Amin et al., 2011, Gammons et al., 2013, Nowak et al., 2010). VEGF-A_xxx_a isoforms are widely known as pro-angiogenic/cytoprotective factors and this splicing pathway is strongly associated with solid tumor development (Amin et al., 2011). Peripheral administration of VEGF-A_165_a resulted in pain, as did, somewhat surprisingly, VEGFR2 blockade (Hulse et al., 2014). These findings are supported by observations that systemic VEGF-A receptor blockers result in pain in clinical studies (Burger et al., 2007, Langenberg et al., 2011) and painful experimental neuropathy (Verheyen et al., 2012). In contrast, given intrathecally, the VEGF-R2 antagonist, PTK787 decreased hypersensitivity in naïve and neuropathic rodents (Fig. 8, and Liu et al., 2012), but VEGF-A_165_a again increased hypersensitivity in naïve (Fig. 8) and spinal cord injury rats (Nesic et al., 2010). This latter increase in pain was associated with aberrant myelinated fiber sprouting in dorsal horn and dorsal columns that may be VEGF-A dependent (Nesic et al., 2010). In contrast, van Neervan and colleagues (van Neerven et al., 2010) found only very small anti-nociceptive effects of intrathecal VEGF-A_165_a on pain, and no effect on neuronal function. Observed differences in VEGF-A effects could be attributable to different concentrations used, the source of VEGF-A_165_a, the degree of injury, or different endogenous isoform complement (Bates et al., 2002a). Clinically, elevated levels of VEGF-A in the spinal cord of neuropathic pain patients correlate with reported pain (McCarthy and McCrory, 2013). VEGF-A and VEGF-A receptor 2 are present in both peripheral and central nervous systems including spinal cord (Bates et al., 2002b, Beazley-Long et al., 2013, Sondell et al., 2000). rhVEGF-A_165_a has consistent pro-nociceptive actions peripherally (Hulse et al., 2014) and centrally, and our findings demonstrate that the different VEGF-A isoform subtypes have opposing actions on nociception in the spinal cord, as they do in the periphery (Hulse et al., 2014). We are the first to show that the alternatively spliced isoform, VEGF-A_165_b has anti-nociceptive actions in the spinal cord.

Taken together our observations of: increased spinal splicing factor expression, increased spinal pro-nociceptive VEGF-A_165_a but unchanged VEGF-A_165_b expression, and blockade of pain behavior and VEGF-A expression changes by SPRK1 inhibition, suggest that exogenous and endogenous VEGF-A isoforms modulate spinal nociceptive processing in naïve animals and after peripheral nerve injury. The sites of ligand/receptor expression, the differences in peripheral and central administration, and the current clinical use of many anti-VEGF treatments to treat varied diseases highlight the importance of recognizing the different functions and sites of action of the alternative VEGF-A isoforms.

### 4.4 Myelinated afferents and neuropathic pain

We found that VEGFR2 blockade resulted in inhibition of A fiber nociceptor-mediated nociception, suggesting that endogenous VEGF is involved in spinal processing of A fiber nociceptor inputs. Irrespective of the animal model or human condition of neuropathic pain, the prevailing evidence is that afferents are sensitized (Djouhri et al., 2006, Hulse et al., 2010) both C-fiber (Ali et al., 1999, Chen and Levine, 2007, Djouhri et al., 2006, Khan et al., 2002, Kirillova et al., 2011, Serra et al., 2012, Serra et al., 2014, Shim et al., 2007, Zhu and Henry, 2012) and A-fiber nociceptors (Ueda, 2006, Zhu and Henry, 2012), increasing the afferent barrage to the spinal cord through enhanced stimulus-evoked responses and/or increases in spontaneous/ongoing firing. Other mechanisms, such as neuro-immune interactions, can also contribute to changes in spinal excitability (Uceyler and Sommer, 2008). The result of increased input to and excitability of spinal neurons is central sensitization (Li et al., 1999) leading to hyperalgesia and allodynia. It has been hypothesized that central sensitization allows low threshold A-fiber afferents to “access” pain pathways (Liljencrantz et al., 2013, Tsantoulas et al., 2012) although precise mechanisms are unknown. Early reports of low threshold Aβ fiber mechanoreceptors (LTMs) sprouting into superficial laminae (Woolf et al., 1992) are still debated (Hughes et al., 2003, Woodbury et al., 2008). A-fiber nociceptive afferents, as opposed to LTMs, have similar central terminals in superficial dorsal horn laminae (I and II_o_) in both naïve and nerve injured animals (Woodbury et al., 2008) and may represent the afferents expressing SRSF1. What is clear is that altered central processing of myelinated nociceptor information contributes to neuropathic pain (Molander et al., 1994, Torsney, 2011a, Ziegler et al., 1999b), such as secondary dynamic allodynia (Koltzenburg et al., 1994). Both C-fiber (unmyelinated) and A-fiber (myelinated) pathways can contribute to chronic pain (Liljencrantz et al., 2013, Ziegler et al., 1999b), but this is the first time that VEGFR2 has been implicated in the processing of information in these pathways. If VEGFR2 is involved in A-fiber nociceptive pathways, then this provides a potential new mechanism for the modulation of nociception.

## 5 Conclusion

Here we identify a novel pathway of nociceptive processing through a SRPK1-SRSF1-VEGF-A_xxx_a axis in myelinated nociceptors that is involved in nociception at the level of the spinal cord. During neuropathic pain development SRPK1 drives expression of pro-nociceptive VEGF-A_xxx_a at the level of the spinal cord. Therefore the development of SRPK1 targeted therapy, or other controls for alternative splicing, would be interesting targets for novel analgesic agent development (Donaldson and Beazley-Long, 2016). These findings highlight the importance of understanding control of RNA function, including alternative splicing in relation to pain, and considering specific interactions of splice factors in excitatory networks following peripheral nerve trauma.
